# Plasma CXCL4–DNA/RNA Complexes and Anti-CXCL4 Antibodies Modulation in an SSc Cohort under Iloprost Treatment

**DOI:** 10.3390/reports7030066

**Published:** 2024-08-02

**Authors:** Anna Mennella, Katia Stefanantoni, Raffaella Palazzo, Giuseppe Ocone, Immacolata Pietraforte, Simona Truglia, Ilaria Bisconti, Alba Pisacreta, Valeria Riccieri, Roberto Lande, Loredana Frasca

**Affiliations:** 1 National Center for Global Health, Istituto Superiore di Sanità, Viale Regina Elena 299, 00161 Rome, Italy; anna.mennella@iss.it (A.M.); raffaella.palazzo@iss.it (R.P.); giuseppe.ocone@iss.it (G.O.); alba.pisacreta@iss.it (A.P.); 2 Department of Clinical, Internal, Anesthesiological and Cardiovascular Sciences, Sapienza University of Rome, Viale del Policlinico 155, 00161 Rome, Italy; katia.stefanantoni@uniroma1.it (K.S.); dott.trugliasimona@gmail.com (S.T.); ilaria.bisconti@uniroma1.it (I.B.); valeria.riccieri@uniroma1.it (V.R.); 3 Department of Oncology and Molecular Medicine, Istituto Superiore di Sanità, Viale Regina Elena 299, 00161 Rome, Italy; immacolata.pietraforte@iss.it

**Keywords:** systemic sclerosis (SSc), iloprost, type I interferon (IFN-I), CXCL4, plasmacytoid dendritic cells (pDCs)

## Abstract

Background: Systemic sclerosis (SSc) is an autoimmune disease characterized by vascular and immunity alterations and skin/internal organ fibrosis. Aberrant levels of plasma CXCL4, CXCL4–RNA/DNA complexes, type I IFN (IFN-I) and anti-CXCL4 antibodies characterize SSc. These parameters influence each other: CXCL4–self-DNA/RNA complexes are triggers of IFN-I in plasmacytoid dendritic cells (pDCs), and anti-CXCL4 autoantibodies amplify this effect. Here, we assess the modulation over time of plasma CXCL4 and the related parameters of CXCL4–DNA/RNA complexes, anti-CXCL4 antibodies, IFN-α and TNF-α in an SSc cohort under the synthetic analogue of prostacyclin PGI2 (iloprost) treatment to address contribution of these parameters to pathogenesis and their role as biomarkers. Methods: We analyzed immunological parameters at baseline (T0) and after 3 (T3) and 6 (T6) months in 30 SSc patients. Responders were the patients that lowered their disease activity parameters after six months of treatment. Results: Anti-CXCL4 autoantibodies correlated with both IFN-α and TNF-α levels in SSc plasma. Responders significantly down-regulated serum IFN-α. In seven patients with a shorter disease duration, improvement coincides with a decrease in plasma IFN-α, CXCL4 and TNF-α. Iloprost efficiently blocks pDCs IFN-α production induced by CXCL4–DNA/RNA complexes in vitro. Conclusions: The data suggest a possible role of iloprost as a disease-modifying drug, mainly accompanied by down-regulation of plasma IFN-I levels. Since CXCL4, IFN-I and TNF-α down-modulation was evident and significant in improving SSc patients with a shorter disease duration, these results warrant future investigations on the early use of iloprost to slow SSc progression.

## 1. Introduction

Systemic sclerosis (SSc) is an autoimmune and fibrotic disease with a high disease burden and mortality rate. Inflammation, microvasculopathy and fibrosis are the triad involved in the pathophysiology of SSc, leading to organ failure [1]. Microvascular constriction and endothelial damage (Raynaud’s phenomenon, RP) is the first manifestation of SSc in 90–98% of cases and precedes the disease onset by years, which evolves to a progressive and diffuse fibrosis [2]. The typical features at the nailfold videocapillaroscopy (NVC) and the presence of RP and specific autoantibodies allow identification of a subgroup of patients with early disease (EaSSc: <5 years) [3] who have a high chance to develop SSc [4]. According to an extension of skin fibrosis, it is possible to define two major subsets of SSc: limited cutaneous SSc (lcSSc) and diffuse cutaneous SSc, (dcSSc) [5]. Accumulating evidence now sustains the notion that alteration of both innate and adaptive immunity can lead to the release of an array of inflammatory mediators, such as type I IFN (IFN-I) and platelet factors [2]. In this context, a wide proteomic study showed that the chemokine (C-X-C motif) ligand 4 (CXCL4), also called platelet factor 4 (PF4), a chemokine mainly secreted by activated platelets, is over-expressed in SSc, with the highest plasmatic levels in EaSSc [6]. In the same study, CXCL4 correlates with mRSS (modified Rodnan skin score) and PAH (pulmonary arterial hypertension) [6]. CXCL4 shows pleiotropic inflammatory effects and inhibits angiogenesis [7,8].

Notably, activation of an IFN-I pathway as an early event in SSc is associated, together with high CXCL4, with more severe disease manifestation and poor prognosis [6,9,10,11,12,13]. Our group has uncovered the mechanistic link between CXCL4 and IFN-I production by pDCs: CXCL4 induces IFN-I by pDCs by breaking immune tolerance to self-DNA and self-RNA [12,14]. In detail, CXCL4 forms nanocrystalline complexes with DNA, which enables otherwise inert natural DNA to induce immune amplification via TLR9-activation. Notably, CXCL4–DNA complexes are detectable and measurable in SSc and tend to correlate with the IFN-I signature, especially in eaSSc (eaSSc) [12]. Interestingly, depletion of pDCs in the bleomycin mouse model of SSc reduces inflammation and fibrosis in skin and lung [15], suggesting an essential role of pDCs in SSc. CXCL4–RNA complexes also exist and can stimulate both pDCs [14] and myeloid DC to produced TNF-α and other inflammatory factors [16]: both CXCL4–DNA and CXCL4–RNA complexes stimulate the production of antibodies by memory B-cells [17]. Interestingly, anti-CXCL4 autoantibodies can be generated in SSc patients [17,18,19] and, in vitro, they can further amplify IFN-I production by pDCs [17,18].

At present, drugs able to significantly modify the course of the disease are missing. According to EULAR guidelines, vasoactive therapy, immune-suppressive treatment and symptomatic drugs can be used in SSc depending on the predominant clinical manifestation, with limited controlled data [20]. Despite new promising and very expensive anti-fibrotic drug discovery, the currently available drugs do not arrest disease progression. 

One of the most used drugs to treat the vascular manifestation of SSc is the second-generation synthetic prostaglandin, iloprost, a potent chemically stable analogue of PGI2 [21]. As its natural analogue, iloprost acts on the prostanoid receptors IP and EP and may act directly on some members of the peroxisome proliferator-activated receptor (PPARs) family. Through the differential activation of these receptor families, which are expressed on several hematopoietic and non-hematopoietic cells, iloprost increases cyclic AMP (cAMP) and then regulates vascular tone, inhibits platelet aggregation and participates in many other processes, including those involved in inflammation and fibrosis [22,23]. It has been shown that synthetic prostaglandins may modulate angiogenesis [24,25], have a controversial anti-fibrotic effect via the IL-17 response modulation [26,27,28,29] and have anti-inflammatory effects on myeloid dendritic cells (mDCs) in vitro [30,31]. Only one study addressed the effect of prostaglandin E2 on IFN-I secretion by pDCs stimulated with synthetic TLR9 agonists [32], whereas the role of iloprost as a disease-modifying drug in early SSc patients, together with its capacity to modulate the inflammatory axis CXCL4-IFN-I in vivo and in vitro, is still largely unknown.

The aim of this study was to address the role of CXCL4 and the related factors (anti-CXCL4 antibodies, CXCL4–DNA or CXCL4–RNA complexes, as well as IFN-α and TNF-α) and effects in a longitudinal manner in an SSc cohort treated with iloprost.

## 2. Materials and Methods

### 2.1. Study Design, SSc Patients and Healthy Donors (HD)

Consecutive SSc patients attending the Scleroderma Clinic of Sapienza University of Rome treated with iloprost were enrolled in this study. All the patients fulfilled the ACR/EULAR 2013 [5] criteria for the classification of SSc. Iloprost was given monthly (0.5–2 ng/kg/min/6 h). Age- and sex-matched healthy controls (HD) were also included in the study. We collected the main clinical/demographic data of the patients and HD, as well as their sera, at baseline (T0) and after 3 (T3) and 6 (T6) months. Scleroderma disease activity was measured using clinical disease activity (EScSGAI), and those patients that lowered their EScSGAI at T6 were considered responders.

The study was conducted according to the protocol and good clinical practice principles and the Declaration of Helsinki statements. All the patients gave their informed consent, and the study was approved by the local ethics committee of the AOU Policlinico Umberto I (for SSc patients) and by the ISS ethic committee for the healthy donors (HD) part.

### 2.2. Reagents

CpGA (TLR9 agonist) and R848 (TLR7/8 agonist) were purchased from Invivogen (San Diego, CA, USA), and CXCL4 was synthesized by Biomatik (Wilmington, DE, USA).

Human DNA was extracted [12,14,16]. Human RNA was extracted from peripheral blood mononuclear cells (PBMCs) (from buffy coats) by using an RNeasy Maxi Kit Qiagen, (Dusseldorf, Germany). The resulting RNA was controlled by 2% agarose gel electrophoresis.

### 2.3. ELISA for CXCL4 Measurement Plasma

To measure the CXCL4 plasma of SSc patients and control HD, we used the Human CXCL4/PF4 ELISA from R&D Systems (Minnneapolis, MN, USA). The plasma was diluted 1:100 (after pre-titration experiments) in dilution medium according to each ELISA’s instruction. 

### 2.4. ELISA for CXCL4–DNA and CXCL4–RNA Complex Detection in Plasma

CXCL4–RNA complexes were identified using a capture ELISA. The capturing antibody, 2 μg/mL of a rabbit anti-human CXCL4 antibody was from Abcam (Abcam, Cambridge, UK, cod. AB9561). It was coated to 96-well plates (100 μL) overnight at room temperature. After blocking in PBS 1% BSA (200 μL), the plasma (100 μL diluted to 1:100 in 1% BSA in PBS) was added and incubated for 2 h at room temperature (RT). After incubation, the wells were washed three times with 200 μL of 0.05% Tween 20 in PBS, and a mouse anti-anti-RNA antibody (Novus Biological, Minneapolis, MN, USA, cod NB100-662,1:300) was added for 1 h at room temperature, followed by a goat anti-mouse antibody conjugated with HRP (Sigma-Aldrich, Darmastadt, Germany, 1:1000). After washing 4 times, the chromogenic substrate 3,3′,5,5′-tetramethylbenzidine (TMB) was added and incubated in the dark; the absorbance was measured at 450 nm after stopping the reaction using 2 N HCl. The same procedure was used for detecting the CXCL4–DNA complexes, where the secondary antibody was represented by a mouse anti-dsDNA (Abcam, 1:300). The plasma samples were considered positive when the OD was above an established cut-off, which was calculated as the mean plus two times the standard deviation of the OD values obtained with the HD plasma.

### 2.5. ELISA for IFN-α and TNF-α Measurement in Plasma

The plasma of the SSc patients was diluted 1:4 in phosphate buffer solution (PBS) to assess the presence of IFN-α and TNF-α; both IFN-α and TNF-α were determined by an ELISA kit MabTech (Cincinnati, OH, USA).

### 2.6. Detection of Anti-CXCL4 Antibodies in Plasma

For anti-CXCL4 antibody detection, we used a home-made ELISA, which is described in [17,18]. Briefly, 96-well flat-bottom plates (non-binding surface polystyrene, Corning, NY, USA) were coated with 2 µg/mL CXCL4 in carbonate buffer (0.1 M NaHCHO_3_, pH 9) for 2 h (or overnight) and washed four times with PBS + 0.1% Tween-20. This washing buffer was used for washing at all steps. A blocking buffer containing 2% bovine serum albumin (BSA, Sigma-Aldrich, St. Louis, MO, USA) in PBS was used for at least 1 h (or overnight) to saturate unspecific binding sites. After washing, the plasma was diluted 1:100 in PBS + 2% BSA, followed by a 1-h incubation with a horseradish peroxidase (HRP)-conjugated goat anti-human IgG (Sigma-Aldrich, St. Louis, MO, USA) diluted 1:5000 in PBS. The color was developed for 5 min with 3,3′,5,5′-tetramethylbenzidine (TMB) substrate (Sigma-Aldrich). The reaction was stopped by adding 50 µL of 2 N H_2_SO_4_, and the absorbance was determined at 450 nm with a reference wavelength of 540 nm. Anti-CXCL4 were considered positive and significant when they exceed the mean OD values obtained with healthy donors (HD) plus two standard deviations (SD).

### 2.7. PDC Isolation and Stimulation

Buffy coats were obtained from the Blood Center, Policlinico Umberto I, Rome, IT. After separation of the mononuclear cells by Ficoll (GE Healthcare, Westborough, MA, USA) centrifugation, the pDCs were purified as described [12] using a Diamond Plasmacytoid Dendritic Cell Isolation Kit (Miltenyi Biotec, Bergisch Gladbach, Germany) to obtain 99% purity. The pDCs were stimulated with CXCL4 (1 µM) and huDNA (10 µg/mL) or hRNA (15 µg/mL), which were premixed for 20 min at RT before stimulating the pDCs. Iloprost was added to the pDC at different concentrations in culture plates for 20 min before adding the CXCL4–DNA or CXCL4–RNA complexes.

### 2.8. Statistical Analysis

Differences between the mean values were assessed by Student’s *t* test for unpaired samples or Mann–Whitney to compare. Pearson’s or Spearman’s correlation coefficient was used to assess the correlation between IFN-α levels and levels of CXCL4, CXCL4–DNA/RNA complexes or CXCL4 or antibodies to CXCL4 and the levels of TNF-α with all the same parameters. The statistical significance was set at *p*  <  0.05. Multiple correlations among the experimental parameters assessed in the SSc cohort were analyzed by principal component analysis (PCA) in R using the package “FactoMineR”. From the graphical point of view, the correlation circle, also known as a variable correlation plot, shows the relationships among all the variables. It can be interpreted as follows: positively correlated variables are grouped together, negatively correlated variables are positioned on opposite quadrants of the plot origin, and variable distances and the origin measure the quality of the variables on the factor map. 

## 3. Results

### 3.1. SSc Patients Show Up-Regulation of CXCL4 and Related Parameters Compared to Healthy Donors (HD)

We enrolled 30 SSc patients (M/F = 1/29), with a mean age of 58.2 years and a mean disease duration of 153.6 months. Fifty per cent of the patients were under DMARDs treatment at a stable dose at least 4 months before enrollment in the study. Table 1 shows the main clinical–demographic characteristics of the patients. Sixteen patients were considered responders and fourteen were non-responders.

**Figure 1:**
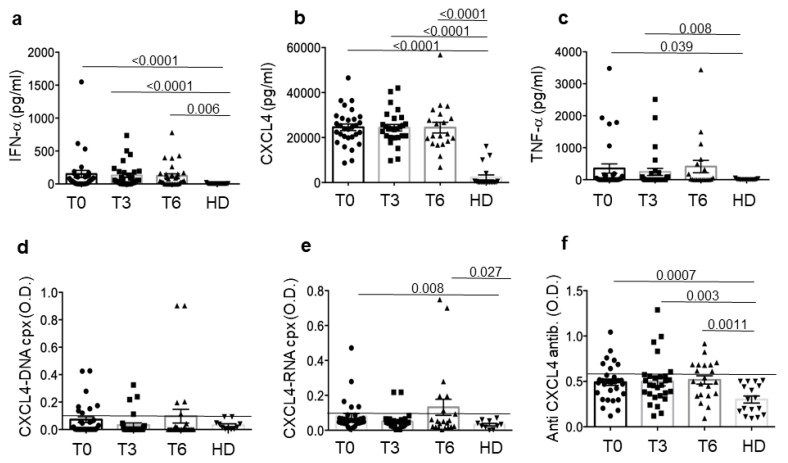
CXCL4-related parameters in the SSc plasma. IFN-α (**a**), ΤΝF−α (**c**), CXCL4 (**b**), immune complexes CXCL4–DNA (**d**) and CXCL4–RNA (**e**) and anti-CXCL4 (**f**) autoantibodies were determined by ELISA assay in SSc patients (N = 30) and healthy donors (HD, N = 17) before treatment (T0), at 3 months (T3) and 6 months (T6). Horizontal bars are the means; vertical bars are the standard error of the mean (SEM); *p*-values are calculated by paired Wilcoxon signed rank test. The mean plus 2 SD (standard deviation) of antibody reactivity or positivity for CXCL4–DNA or CXCL4–RNA of HD was used as cut-off (black line in panels (**d**–**f**)).

We assessed CXCL4, CXCL4–DNA and CXCL4–RNA complexes, anti-CXCL4 autoantibodies and IFN-α and TNF-α, which we call “CXCL4-related parameters” by ELISA at base line (T0) and at two different time points of treatments (three months, T3, and six months, T6) in SSc patients and in 19 HD (matched for age and sex). All the parameters were mostly significantly upregulated in SSc plasma as compared to HD plasma (Figure 1) or showed a tendency to be higher in SSc than in HD. Plasma CXCL4 was higher in early SSc (eaSSc, disease duration < 5 years) than in long-lasting SSc (lsSSc, disease duration > 5 years) (Figure S1). The results confirm that CXCL4 is overexpressed [6,12,33] and is an autoantigen in SSc, as anti-CXCL4 autoantibodies were detected in several SSc patients [17,18]. The presence of plasma CXCL4–DNA and CXCL4–RNA complexes and IFN-α and TNF-α expression are also expressed in SSc blood, as observed in other studies [12,16,17]. Interestingly, anti-CXCL4 antibodies correlated with IFN-α and TNF-α in the SSc plasma in our SSc cohort (anti-CXCL4 at T0 antibodies versus IFN-α at T0: r = 0.33, *p* = 0.038, *n* = 30; anti-CXCL4 at T0 versus TNF-α at T0: r = 0.33, *p* = 0.036, *n* = 30, by Spearman’s correlation test). 

Overall, these results confirm previous observations and reveal that all the CXCL4-related parameters measured were up-regulated and remained up-regulated over time in SSc patients as compared to HD.

### 3.2. SSc Responders Reduce IFN-I Plasma Levels and TNF-α

The results presented above prompted us to address more in detail the way in which all the parameters were modulated over time during therapeutic protocols that included iloprost. Indeed, iloprost is a modulator of platelet-derived factors and IFN-I. 

**Figure 2:**
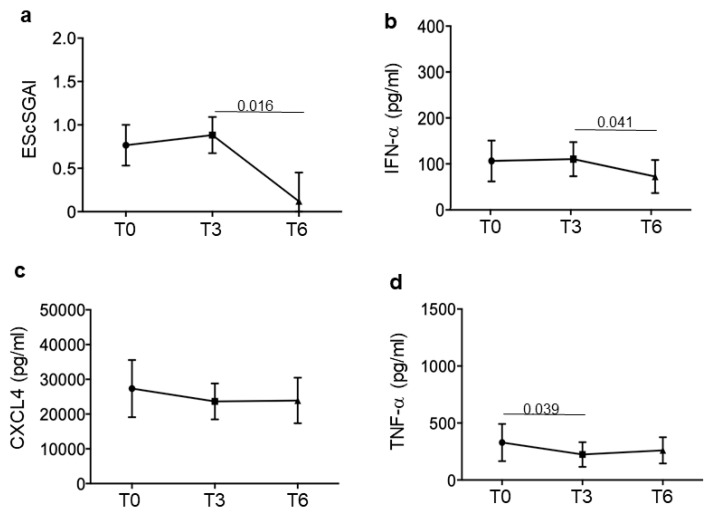
CXCL4-related parameters in responder SSc patients. Disease activity score (EScSGAI, (**a**)) and plasma IFN-α (**b**), CXCL4 (**c**) and TNF-α (**d**) were measured in responder patients (N = 16) at the time points in Figure 1. Data are plotted as mean plus standard error of the mean (SEM); *p*-values are calculated by paired Wilcoxon signed rank test.

**Figure 3:**
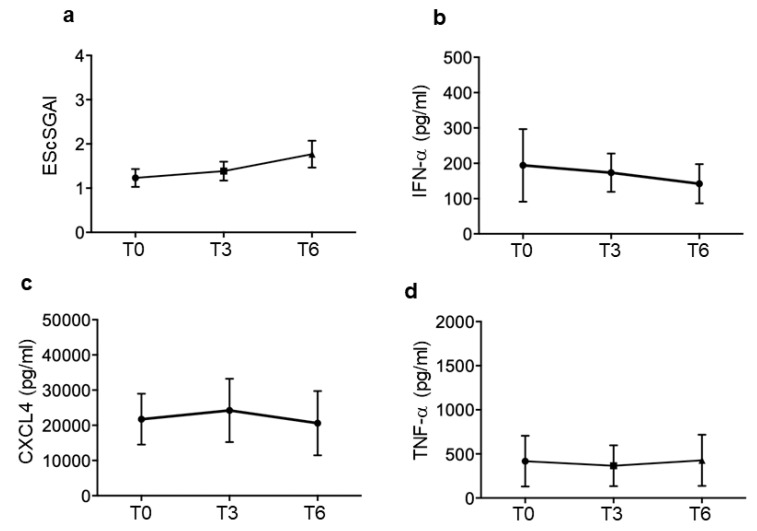
CXCL4-related parameters in non-responder SSc patients. Disease activity score (EScSGAI, (**a**)) and plasma IFN-α (**b**), CXCL4 (**c**) and TNF-α (**d**) were measured in non-responder patients (N = 14) at the time points in Figure 1. Data are plotted as mean plus standard error of the mean (SEM); *p*-values are calculated by paired Wilcoxon signed rank test.

To obtain a clearer picture, we divided our SSc patients in responders and non-responders to treatment. Responders had minimal disease activity, measured as EScSGAI, which did not increase over time, or a higher disease activity, which decreased at T6 (six months treatment) (Figure 2a) but not necessarily at T3 (some of the patients slightly increased their EScSGAI at T3 but they decreased it at the end of the observation period). Non-responders were those SSc patients that did not lower or even increased their EScSGAI at T6 (Figure 3a). Figure 2 and Figure 3 show the mean (plus/minus standard error of the mean) of the EScSGAI and the expression levels of CXCL4, IFN-α and TNF-α in 30 SSc patients at baseline (T0, before treatment) and after 3 and 6 months of treatment. Only in the responders (N = 16; Figure 2), but not in the non-responders (N = 14; Figure 3), was a significant reduction in the EScSGAI at T6 paralleled by significant down-regulation of plasma IFN-α between T3 and T6 (*p* = 0.041) (Figure 2b). A certain decrease in TNF-α was also observed in the responders (Figure 2d). The other related parameters (CXCL4-DNA and CXCL4–RNA complexes, as well as anti-CXCL4 antibodies) were not significantly modulated over time (Figure S2).

These results suggest that a decrease in IFN-I and TNF-α in blood and disease improvement, measured by a reduction in the EScSGAI, may be linked phenomena.

### 3.3. In the Non-Responders, CXCL4 and All Related Parameters Correlate with the EScSGAI

We used a multi-parametric PCA analysis (see Section 2) to better understand the reciprocal distribution and correlation among CXCL4 and CXCL4–DNA/RNA complexes, as well as with the anti-CXCL4 antibodies, which are all factors that may influence the IFN-α and TNF-α induction and may play a role in disease [12,16,17]. In the entire cohort, the relationship between the presence of circulating anti-CXCL4 antibodies and plasma levels of IFN-α or TNF-α, (assessed by Spearman’s correlation test as in Section 3.1) was confirmed by this analysis, depicted in Figure S3. The positive correlation between anti-CXCL4 antibodies and TNF-α or IFN-α is indicated by the acute angle in the upper-right quadrant (see Section 2), between the anti-CXCL4 antibodies arrow and arrows representing each of these cytokines. 

In the cohort as a whole, it was not evident if there was a significant correlation between each of the parameters tested and the disease activity. 

**Figure 4:**
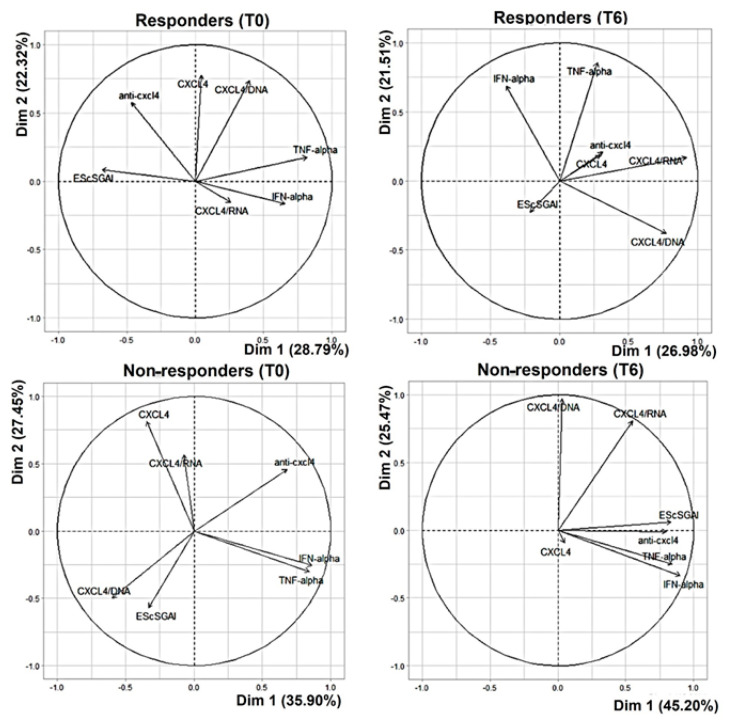
Principal component analysis plot of responder and non-responder SSc patients. Correlation circles of 7 variables and the first and second dimensions of responder patients (N = 16) at baseline (T0; 51% of the total variance explained) and at 6 months (T6; 48.5% of the total variance explained) and of non-responder subjects (N = 14) at baseline (T0; 63.5% of the total variance explained) and at 6 months (T6; 70.6% of the total variance explained).

We sorted the SSc patients into responders and non-responders, and we looked at the situation at T0 in these two groups and at the end of the observation period, T6 (Figure 4). The EScSGAI correlated with anti-CXCL4 antibodies in the responders, whereas in the non-responders, there were anti-CXCL4–DNA complexes that correlated with the EScSGAI (the correspondent statistical values are reported in Tables S1 and S2). Moreover, the same Tables S1 and S2 report a chart with the entire “cross-correlations” among all the parameters tested (significance is reported where present), which have been analyzed by PCA.

This may indicate that autoantibodies may have a more pronounced pathogenic role in the responders. 

The pictures show some additional correlations, in particular, between TNF-α and IFN-α in both groups at T0, and between CXCL4–DNA complexes and TNF-α and IFN-α in the responders.

Of interest, the CXCL4–DNA and CXCL4–RNA complexes were in correlation with IFN-α and TNF-α in the responders, but these correlations diminished for the CXCL4–RNA complexes and were absent for the CXCL4–DNA complexes at T6. This suggests that CXCL4–DNA complexes no longer contribute to the IFN-I signature after disease improvement (T6). The CXCL4–RNA complexes also diminished their contribution to the same signature.

When the responders lowered their EScSGAI (not all patients reached a value of zero for the EScSGAI), none of the assessed parameters remained in correlation with the EScSGAI in the group of responders (Figure 4, responders T6). Vice versa, in the non-responders, all the assessed parameters, with variable intensity, remained in strict correlation with the EScSGAI and with each other (Figure 4, non-responders at T6). The parameter that remained less correlated with the EScSGAI in the non-responders was the presence of blood CXCL4–DNA complexes. This result suggests that such parameters might no longer play a relevant role in the disease after treatment. 

### 3.4. Iloprost Treatment Reduces Disease Activity in an SSc Subgroup with a Shorter Disease Duration

**Figure 5:**
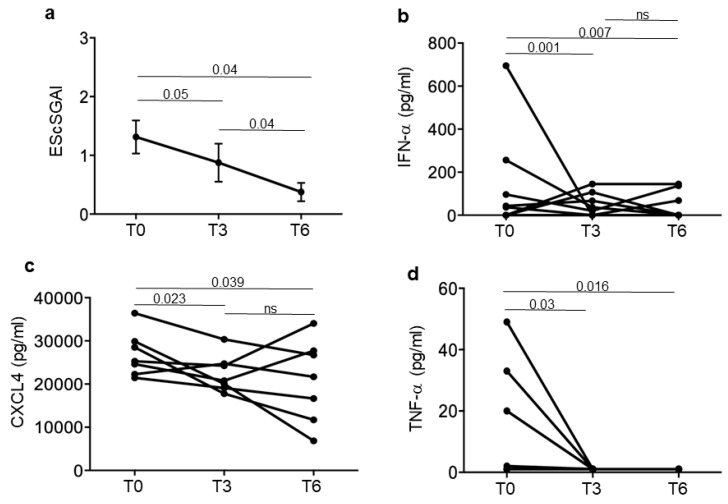
Disease activity and CXCL4-related parameters in eaSSc patients. Disease activity score (EScSGAI, (**a**)) and plasma IFN-α (**b**), CXCL4 (**c**) and TNF-α (**d**) were measured in early patients (N = 7) at the indicated time points. Data are plotted as mean plus standard error of the mean (SEM); *p*-values are calculated by paired Wilcoxon signed rank test.

We observed a significant EScSGAI decrease at T6 in a sub-group of patients (N = 7) (Figure 5a). Interestingly, this latter sub-group responded to the definition EaSSc. In these patients, we observed a significant decrease in IFN-α and TNF-α already at T3, which persisted at T6 (Figure 5b,d). Moreover, a decrease at T3 was also observed for the CXCL4 blood levels, although not all the patients had the same behavior regarding CXCL4 expression (Figure 5c). All the other parameters (autoantibodies to CXCL4 and immune complexes of CXCL4 with DNA or RNA) were not significantly modulated in these 7 patients (Figure S5). 

These results indicate that a decrease in EScSGAI can be preceded by an early and persistent plasma IFN-I (and in TNF-α) reduction in eaSSc. 

**Figure 6:**
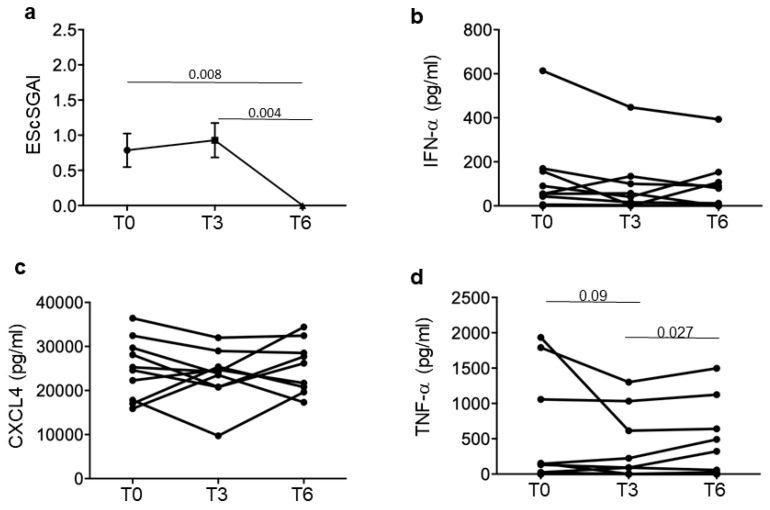
Disease activity and CXCL4-related parameters in the responders with long-lasting SSc. Disease activity score (EScSGAI, (**a)**) and plasma IFN-α (**b**), CXCL4 (**c**) and TNF-α (**d**) were measured in the responder patients (N = 9) with a disease duration > 5 years at the indicated time points. Data are plotted as mean plus standard error of the mean (SEM); *p*-values are calculated by paired Wilcoxon signed rank test.

We also present the same data in the remaining 9 SSc patients that also responded to treatment but do not belong to the group of EaSSc and have a variable disease duration > 5 years (Figure 6). In these patients, a tendency towards a decrease in IFN-α and TNF-α is also apparent, although it is not statistically significant. 

Overall, these results suggest that the eaSSc responds to therapy in a better manner compared to the long-lasting SSc.

### 3.5. Iloprost Impairs pDCs Activation in the Presence of CXCL4–DNA/RNA Complexes

**Figure 7:**
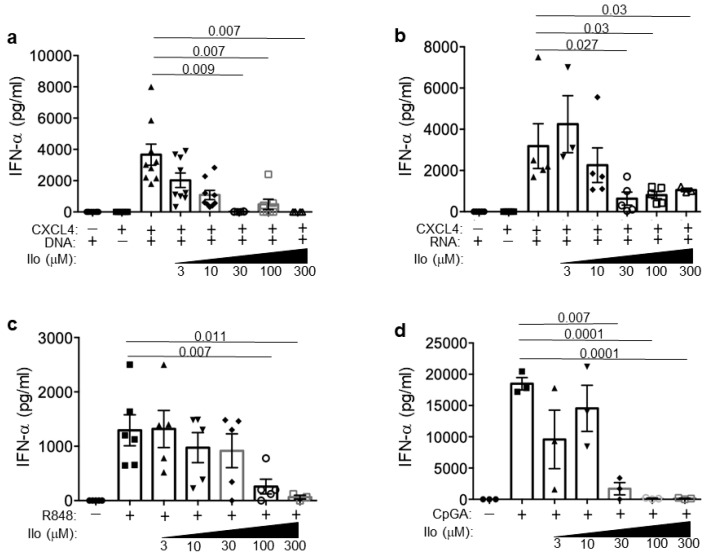
Iloprost reduces TLR-triggered pDC-derived IFN-α in vitro. IFN-α released in the supernatant from healthy pDCs stimulated with immune complexes CXCL4–DNA (**a**) and CXCL4–RNA (**b**), or TLR7/8 ligand R848 (**c**) and TLR9 ligand CpGA (**d**) alone or in the presence of the indicated doses of iloprost at 24 h. IFN-α content was measured by ELISA assay. Horizontal bars are the means; vertical bars are the standard error of the mean (SEM); *p*-values are calculated by two-tailed Student’s *t* test for paired samples from 5–8 different donors.

The responder patients experienced a decrease in their IFN-I signature. To experimentally explore whether iloprost may result in the IFN-I inhibition, we used iloprost in vitro on pDCs, as these cells are the main IFN-I producers and play a role in SSc. We isolated pDCs from buffy coats (see Section 2) and stimulated them in vitro with CXCL4–DNA and CXCL4–RNA complexes in the absence or in the presence of scalar doses of iloprost (Figure 7). The results show that the capacity of CXCL4–DNA complexes to stimulate pDC-derived IFN-α was dose-dependently reduced in the presence of iloprost (Figure 7a). This inhibitory effect was seen also upon CpGA stimulation (as a control). A slightly lower but similar effect was seen in response to the CXCL4–RNA complex stimulation of R848 (a TLR7/8 agonist).

Thus, the interferogenic effect of the CXCL4–DNA/RNA complexes was decreased in the presence of iloprost, which may possibly mimic the effect that iloprost may have in vivo, namely, the blocking of the IFN-I pathway activation likely mediated by these complexes.

## 4. Discussion

In this pilot study, we analyzed a series of experimental immunological parameters linked to CXCL4, an important biomarker in SSc [6], in an SSc cohort treated with iloprost (and standard therapy; see Table 1), one of the most commonly used drugs that treats the vascular manifestation of SSc [21].

This is the first study that loingitudinally analyzes all these CXCL4-related experimental parameters (not routinely assessed in the clinical settings at the moment), which are strictly linked to the IFN-I signature, in an SSc cohort. The cohort was treated with iloprost, a synthetic prostaglandin that is a potent, chemically stable analogue of PGI2, synthesized by Schering AG in 1978. Standard immunosuppression was also used in half of the patients, but this was pre-existing and stable for three months before starting iloprost, so we expect that this does not interfere significantly with the situation observed at the time points of the study. Iloprost regulates vascular tone, acting as a dilator of vascular smooth muscle. Importantly, iloprost acts as a potent inhibitor of platelet aggregation [22,23,34], and this action could explain the tendency, seen in our responder patients with eaSSc, towards a reduction in blood CXCL4, which is a platelet product [7], with a parallel improvement of the disease activity (EScSGAI). This reduction was evident in a small group of eaSSc patients already after three months of treatment. In all the 16 SSc patients responsive to treatment, the typical IFN-I signature often detected in SSc patients was also significantly reduced when the disease index (EScSGAI) dropped. 

Besides the reported clinical observations, we provide in vitro data that demonstrate that iloprost is able to dose-dependently inhibit IFN-α induced by CXCL4–DNA and CXCL4–RNA complex stimulation in pDCs. This capacity could be instrumental in improving disease activity in SSc. This is the first demonstration that iloprost can inhibit the IFN-α production induced by CXCL4 in complex with DNA and RNA. Only one paper, so far, has shown that iloprost could inhibit the activation of pDCs, but in that paper, a synthetic compound, CpGA, was used to stimulate the cells rather than natural ligands like the CXCL4–DNA complexes [32]. CXCL4–RNA complex stimulation of pDCs was also lowered by iloprost, showing that activation via TLR7 was also impaired. The fact that iloprost works by inhibiting pDC activation is of interest, as pDCs are crucial players in the pathogenesis of SSc [35]. 

The in vitro data seem in keeping with the results of the PCA analysis at T6 in the responders: in these patients, the CXCL4–DNA complexes did not disappear from circulation over time, but they lost their relationship with IFN-α up-regulation, which may reflect the capacity of iloprost in vivo, like that observed in vitro, to inhibit pDC activation by these complexes. These results suggest that iloprost may be useful to decrease the two important factors that are interconnected, CXCL4 and IFN-I. CXCL4 forms nanocrystalline complexes with DNA, which enables self-DNA to induce immune amplification via TLR9-activation [12,36]. CXCL4 also works as a “danger signal” in complex with RNA [16,37]. The SSc patients responsive to the therapy could thus be those patients in which iloprost has success in blocking pDC activation by these complexes. It is important to underlie that activation of an IFN-I pathway as an early event in SSc is associated with more severe disease manifestation and poor prognosis [9]. High circulating levels of CXCL4 in early disease stages are associated with poor disease prognosis, increased fibrosis and higher lung involvement [6,11,12]. Our findings suggest that eaSSc patients may be those that could better benefit from the timely use of iloprost to block the CXCL4 activity (in complex with nucleic acids) and IFN-I linked pathways: early treatment may translate into a slower progression of the disease and fewer complications afterwards. This is something that could be addressed by administering iloprost at early SSc stages, and then following patients over a relatively long period of time.

Our PCA analysis shows interconnections among the parameters analyzed and reveals that patients that improved have a disease activity that is no longer in correlation with this same “CXCL4-related parameters”. 

Improvement, in our cohort, seems to also be associated with some TNF-α inhibition. This may be also linked to iloprost treatment, in that it has been shown that iloprost exerts an anti-inflammatory effect on myeloid DCs, seen as inhibition of TNF-α (among other factors) release by these cells upon iloprost treatment [30,38]. This anti-inflammatory activity can suppress T-cell activation or, as seen in a mouse model, induce regulatory T-cells [38,39].

Our analysis also reinforces the potential role of anti-CXCL4 autoantibodies in SSc. They correlated with disease activity at T0 in the responders, and this correlation was lost at T6. We have demonstrated that such antibodies can further implement IFN-α production by pDCs [17]. However, we expect that this amplification is also blocked by iloprost. Anti-CXCL4 autoantibodies remain in strong correlation with the disease activity and IFN-α and TNF-α in the non-responders, which reinforces the idea that these antibodies may be pathogenic via IFN-I amplification. In addition, though, they may contribute to forming immune complexes with several harmful effects. For instance, they can favor neutrophil extracellular traps formation (NETosis), a phenomenon that may occur in SSc [40].

This study has several limitations. First of all, we do not have a control cohort untreated with iloprost. Second, only one patient had an active disease (EScSGAI>3) in this cohort (at visit) and we cannot address whether an active disease further affects the parameters measured in this study. Third, we cannot presently explain why the iloprost treatment does not inhibit, or at least limit, IFN-α expression in all the treated patients’ blood. The PCA suggests that CXCL4–DNA complexes strongly correlated with the disease activity in the non-responders more than in the responders. These complexes could be more stimulatory in the non-responders, and iloprost may not be sufficient to block their effect. However, at T6, the presence of these CXCL4–DNA complexes seems to dissociate from the IFN-I production (these complexes no longer correlate with IFN-α at T6). In addition, they represent the sole parameters that correlate less with the disease activity at T6. It could be inferred that additional pathogenic mechanisms may be at work in the non-responders, which do not allow blocking of the IFN-I signature. In this regard, it is worth noting that the final EScSGAI in the non-responders (at T6) remains in strong correlation with the anti-CXCL4 antibodies in plasma, which, in turn, strongly correlate with IFN-α and TNF-α. Thus, anti-CXCL4 antibodies can possibly change their role in the course of the disease and become more pathogenic; this is something that can be explored in the future.

Further studies are needed to evaluate the exact impact of CXCL4 and the related parameters studied here (including autoantibodies) on the fibrotic manifestations in SSc patients. Wider studies can also analyze the same parameters in wider SSc cohorts.

## 5. Conclusions

This study is a first attempt to analyze the longitudinal modulation of CXCL4 and the related parameters of CXCL4–DNA/RNA complexes and anti-CXCL4 autoantibodies, as well as their relationship with IFN-α/TNF-α in an SSc cohort under iloprost treatment. This study shows that all the parameters analyzed can have a relationship with disease activity, and larger studies may find them to predict a response to therapies. The data suggest that early iloprost treatment is more efficacious in blocking disease activity and, likely, progression, although more dedicated studies in larger early patient cohorts are needed.

## Figures and Tables

**Table 1:** Main clinical–demographic and laboratory characteristics of the SSc patient cohort.

**Demographic Data:**	**Patients** N = 30
Sex (M/F)	1/29
Mean age in years (range)	58.2 (37–79)
Mean disease duration in months (range)	153.6 (12–468)
SSc subtype (lSSc/dSSc)	18/12
**Clinical and Laboratory Features N/%**	
Raynaud’s phenomenon	30/100
Digital ulcers	20/66.6
Telangiectasia	20/66.6
Calcinosis	6/20
Lung fibrosis	14/46.6
Pulmonary arterial hypertension	0/0
Gastro-intestinal involvement	22/73.3
Articular involvement	15/50
Cardiac involvement	10/33.3
mRSS ≥ 14	9/30
ANA+	30/100
Anti-Scl70+	17/56.6
ACA+	6/20
EaSSc/long-lasting SSc	7/23
Mean EScSGAI T0/T6	1.18/1.05
**Concomitant Drugs N/%**	
DMARDs	15/50
Methotrexate	8/26.6
Azathioprine	4/13.3
Rituximab	1/3.3
Hydroxychloroquine	2/6.6
Vasoactive drugs	29/96.6
Bosentan	9/30
Sildenafil	4/13.3
Calcium channel blockers	24/80
Pentoxyfilline	5/16.6
Glucocorticoids	17/56.6

Abbreviations: lSSc/dSSc: limited/diffuse systemic sclerosis; mRSS: modified Rodnan skin score; EScSGAI: European Scleroderma Study Group Activity Index; ANA: antinuclear antibodies; anti-Scl70: anti-topoisomerase 1 antibodies; ACA: anti-centromere antibodies; DMARDs: disease-modifying antirheumatic drugs. Only one patient had active disease (ESCsGAI > 3) at T0 and T6.

## Data Availability

The original data presented in this study are available on reasonable request from the corresponding authors. The data are not publicly available due to privacy.

## Supplementary Materials

The following supporting information can be downloaded at: https://www.mdpi.com/article/10.3390/reports7030066/s1, Figure S1: Circulating CXCL4 in sub-groups of SSc patients stratified for disease duration. Figure S2: ELISA measurement of CXCL4–DNA/RNA complexes and anti-CXCL4 autoantibodies in SSc responders and non-responders. Figure S3: Principal component analysis plot of all SSc patients. Figure S4: Circulating CXCL4–nucleic acid immune complexes and anti-CXCL4 autoantibodies of early SSc patients. Table S1: Correlation among CXCL4 and related parameters in SSc responders; Table S2: Correlation among CXCL4 and related parameters in SSc non-responders.
