# Supplementary material for: Plasma CXCL4–DNA/RNA Complexes and Anti-CXCL4 Antibodies Modulation in an SSc Cohort under Iloprost Treatment
Source: Reports (MDPI). 2024 Aug 2;7(3):66. doi: 10.3390/reports7030066 (PMC12225429; doi:10.3390/reports7030066)
Supplement: Supplementary file 1 [file reports-07-00066-s001.zip › reports-3109468-supplementary.pdf]

Figure S1

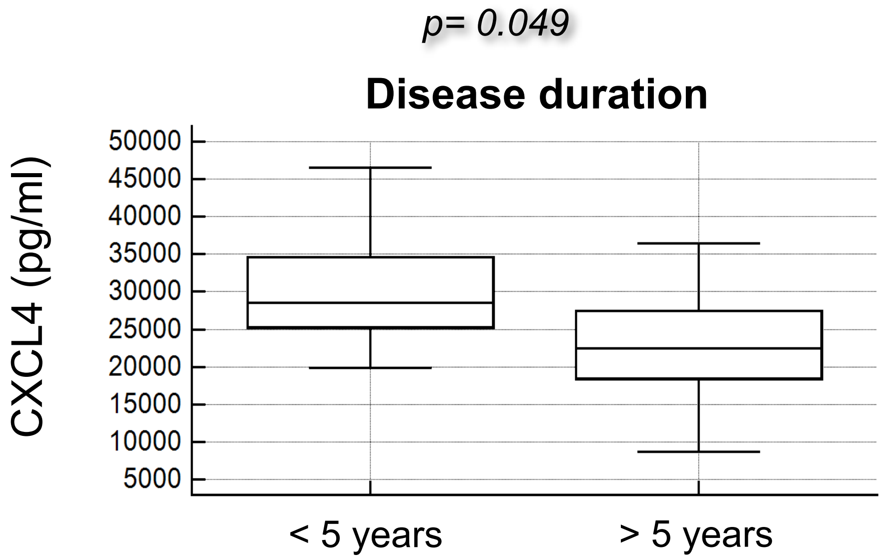

Figure S1. Circulating CXCL4 in sub-groups of SSc patients stratified for disease duration. CXCL4 levels in the plasma of SSc patients with a disease duration < 5 years (N=7) and > 5 years (N=23). CXCL4 content was measured by ELISA assay. Data are plotted as mean plus standard error of the mean (SEM); p-values are calculated by paired Wilcoxon signed rank test.

Figure S2

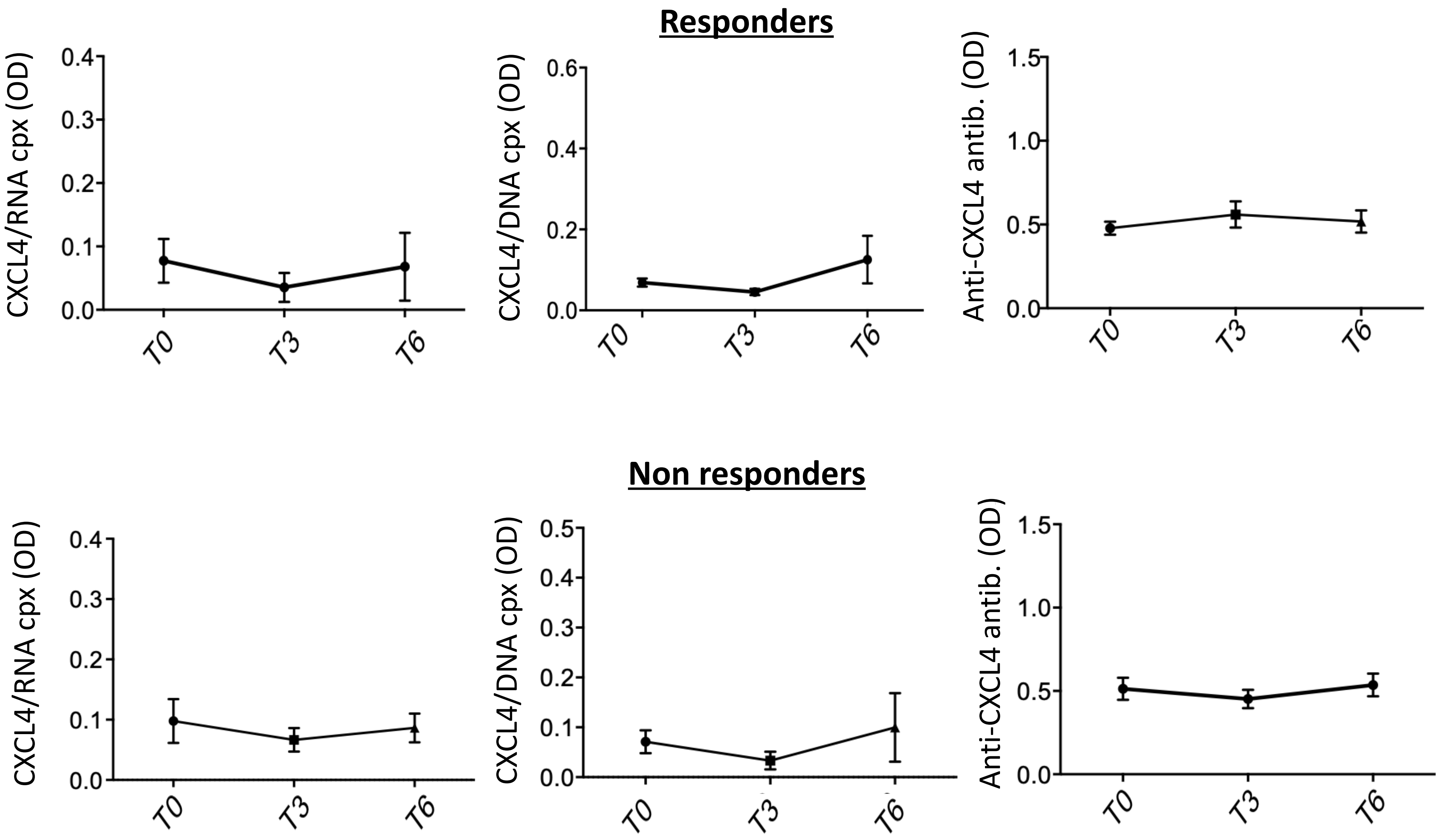

Figure S2. ELISA measurement of CXCL4-DNA/RNA complexes and anti-CXCL4 autoantibodies in SSc responders and non responders. CXCL4-DNA, CXCL4-RNA immune complexes and anti-CXCL4 antibodies were measured by ELISA assay in the plasma of Responder (N=16) and Non Responder patients (N=14) before treatment (T0), at 3 months (T3) and 6 months (T6). Horizontal bars are the means; vertical bars are the standard error of the mean (SEM); p-values are calculated by paired Wilcoxon signed rank test.

Figure S3

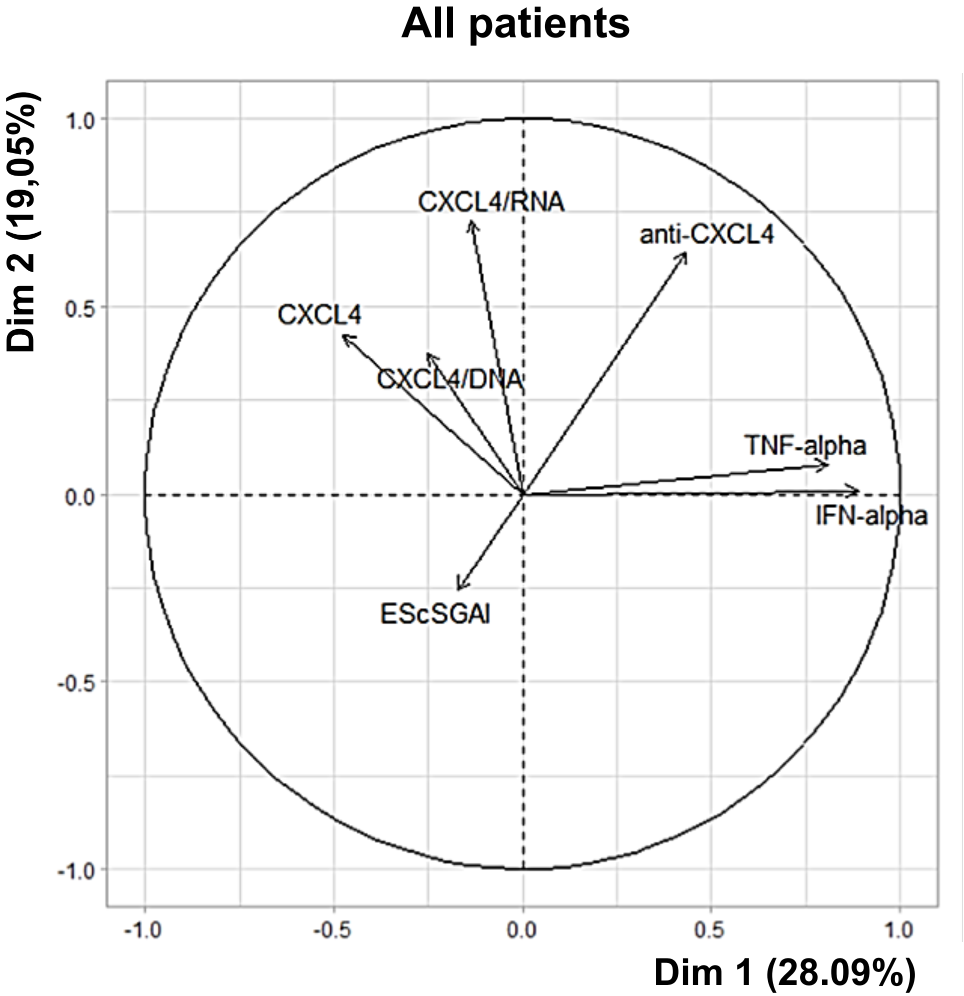

Figure S3. Principal Component Analysis plot of all SSc patients. Correlation circles of 7 variables and the first and second dimensions of all SSc patients (N=30) at baseline (47% of the total variance explained).

Figure S4

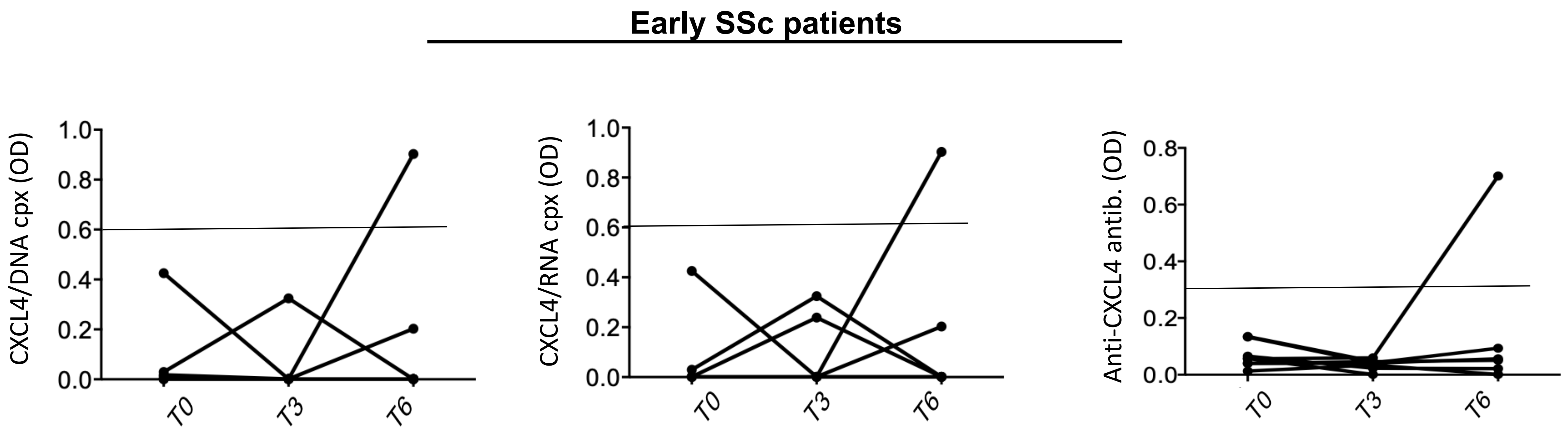

Figure S4. Circulating CXCL4-nucleic acid immune complexes and anti-CXCL4 autoantibodies of early SSc patients. CXCL4-DNA, CXCL4-RNA immune complexes and anti-CXCL4 antibodies were measured by ELISA assay in the plasma of early SSc patients (N=7) before treatment (T0), at 3 months (T3) and 6 months (T6). Horizontal bars are the means; vertical bars are the standard error of the mean (SEM); p-values are calculated by paired Wilcoxon signed rank test.

RESPONDERS (T0)

	EScSGAI	IFN-α	CXCL 4	CXCL4/ DNA	CXCL4/ RNA	TNF-α	anti- CXCL4
**EScSGAI**		r=-0.68 p=0.003 (Dim.1)	NS	NS	NS	NS	r=0.44 p=0.03 (Dim.2)
**IFN-α**	r=-0.68 p=0.003 (Dim.1)		NS	NS	r=0.92 p= 1.50e-05 (Dim.3)	r=0.92 p=7.13e -05 (Dim.1)	NS
**CXCL4**	NS	NS		r=0.85 p=0.001 (Dim.2)	NS	NS	r=0.75 p=0.01 (Dim.2)
**CXCL4/ DNA**	NS	NS	r=0.85 p=0.00 1 (Dim.2)		NS	NS	r=0.67 p=0.06 (Dim.1)
**CXCL4/ RNA**	NS	r=0.92 p= 1.50e-05 (Dim.3)	NS	NS		r=0.90 p= 1.58e-0 5 (Dim.3)	NS
**TNF-α**	NS	r=0.92 p=7.13e-05 (Dim.1)	NS	NS	r=0.90 p= 1.58e-05 (Dim.3)		NS
**anti- CXCL4**	r=0.44 p=0.03 (Dim.2)	NS	r=0.75 p=0.01 (Dim.2)	r=0.67 p=0.06 (Dim.1)	NS	NS	

RESPONDERS (T6)

	EScSGAI	IFN-α	CXCL 4	CXCL4/ DNA	CXCL4/ RNA	TNF-α	anti- CXCL4
**EScSGAI**		NS	NS	NS	NS	NS	NS
**IFN-α**	NS		NS	NS	NS	r=92 p=2.26e-0 5 (Dim.2)	NS
**CXCL4**	NS	NS		NS	NS	NS	r=0.81 p=0.005 (Dim.3)
**CXCL4/ DNA**	NS	NS	NS		r=0,95 p=4.73e- 04 (Dim.1)	NS	r=0.67 p=0.06 (Dim.1)
**CXCL4 /RNA**	NS	NS	NS	r=0,95 p=4.73e- 04 (Dim.1)		NS	NS
**TNF-α**	NS	r=92 p=2.26e-05 (Dim.2)	NS	NS	NS		NS
**anti- CXCL4**	NS	NS	r=0.81 p=0.00 5 (Dim.3)	r=0.67 p=0.06 (Dim.1)	NS	NS	

Table S1. Correlation among CXCL4 and related parameters in SSc responders. Coefficient of correlation r, significance P, and Dimension (Dim.) relative to the PCA analysis of the reported variables (EScSGAI, disease activity; IFN-alpha, CXCL4 and complexes with nucleic acids and anti-CXCL4 antibodies), measured in responders at T0 (baseline) and T6 (after six months treatment) (N=16), by commercial or home-made ELISA. Correlation was calculated by Pearson's correlation test.

NON RESPONDERS (T0)

	EScSGAI	IFN-α	CXCL4	CXCL4/ DNA	CXCL4/ RNA	TNF-α	anti-CXCL4
**EScSGAI**		NS	NS	r=0.7 p=0.03 (Dim.2)	NS	NS	NS
**IFN-α**	NS		NS	NS	NS	r=0.93 p=7.13e-05 (Dim.1)	r=0.81 p=7.63e-03 (Dim.1)
**CXCL4**	NS	NS		NS	r=0,9 p=0.000 3 (Dim.2)	NS	r=0.81 p=0.005 (Dim.3)
**CXCL4/ DNA**	r=0.7 p=0.03 (Dim.2)	NS	NS		r=095 p=4.73e-04 (Dim.1)	NS	r=0.67 p=0.06 (Dim.1)
**CXCL4/ RNA**	NS	NS	r=0,9 p=0.000 3 (Dim.2)	r=0,95 p=4.73e-04 (Dim.1)		NS	NS
**TNF-α**	NS	r=0.93 p=7.13e-05 (Dim.1)	NS	NS	NS		r=0.6 p=1.55e-04 (Dim.1)
**anti-CXCL4**	NS	r=0.81 p=7.63e-03 (Dim.1)	r=0.81 p=0.005 (Dim.3)	r=0.67 p=0.06 (Dim.1)	NS	r=0,6 p=1.55e-04 (Dim.1)	

NON RESPONDERS (T6)

	EScSGAI	IFN-α	CXCL 4	CXCL4/ DNA	CXCL4/ RNA	TNF-α	anti-CXCL4
**EScSGAI**		NS	NS	NS	NS	r=0.89 p=4.74e-04 (Dim.1)	r=0.91 p=2.06e-04 (Dim.1)
**IFN-α**	NS		NS	NS	NS	r=0.94 p=1.09e-05 (Dim.1)	NS
**CXCL4**	NS	NS		NS	NS	NS	NS
**CXCL4/ DNA**	NS	NS	NS		r=0.98 p=7.26e-09 (Dim.2)	NS	NS
**CXCL4/ RNA**	NS	NS	NS	r=0,98 p=7.26e-09 (Dim.2)		NS	NS
**TNF-α**	r=0.89 p=4.74e-0 4 (Dim.1)	r=0.94 p=1.09e-05 (Dim.1)	NS	NS	NS		r=0.9 1.60e-04 (Dim.1)
**anti-CXCL4**	r=0.91 p=2.06e-0 4 (Dim.1)	NS	NS	NS	NS	r=0.9 1.60e-04 (Dim.1)	

Table S2. Correlation among CXCL4 and related parameters in SSc non responders. Coefficient of correlation r, significance P, and Dimension (Dim.) relative to the PCA analysis of the reported variables (EScSGAI, disease activity; IFN-alpha, CXCL4 and complexes with nucleic acids and anti-CXCL4 antibodies), measured in non responders at T0 (baseline) and T6 (after six months treatment) (N=14), by commercial or home-made ELISA. Correlation was calculated by Pearson's correlation test.
